# Immune profiling of ART-conceived children in Kazakhstan: a case-control study

**DOI:** 10.3389/fped.2024.1447956

**Published:** 2024-11-22

**Authors:** Sevara Ilmuratova, Vyacheslav Lokshin, Andrey Prodeus, Lyazzat Manzhuova, Zhanar Nurgaliyeva, Farida Kussainova, Aygul Bazarbaeva, Valeriya Nekhorosheva, Aygerim Abshekenova

**Affiliations:** ^1^ Department of Science, Kazakhstan Medical University “KSPH”, Almaty, Kazakhstan; ^2^ Department of Assisted Reproductive Technologies, International Clinical Centre of Reproduction “PERSONA”, Almaty, Kazakhstan; ^3^ Science and Education Department, International Academy of Reproductology, Almaty, Kazakhstan; ^4^ Science and Education Department, Scientific Center of Pediatrics and Pediatric Surgery, Almaty, Kazakhstan; ^5^ Department of Outpatient Pediatrics, School of Pediatrics, Asfendiyarov Kazakh National Medical University, Almaty, Kazakhstan; ^6^ Department of Gynecology, Asfendiyarov Kazakh National Medical University, Almaty, Kazakhstan; ^7^ Department of Assisted Reproductive Technologies, Institute of Reproductive Medicine, Almaty, Kazakhstan

**Keywords:** assisted reproductive technology (ART), children, immune system, immune dysregulation, C-section=caesarean section, breastfeeding

## Abstract

**Objective:**

The increasing use of assisted reproductive technologies (ART) has led to a growing interest in the health outcomes of offspring. However, the impact of ART on the immune system of children remains poorly understood. While only two publications were found, their findings contradict each other and did not consider other risk factors in their analysis except for ART use. Therefore, this study aimed to examine the potential impact of ART on the immune system of offspring.

**Methods:**

A case-control study was conducted in Kazakhstan to investigate the immune system of ART-conceived children compared to those conceived naturally (NC). The study included participants who met certain criteria, such as having undergone a successful ART program resulting in the birth of either a single or multiple pregnancies. Patients who used donor oocytes/sperm, intrauterine insemination, or surrogacy were excluded. Anamnesis data were collected from children in both groups, and laboratory measurements were performed and analyzed using IBM SPSS Statistic 26.

**Results:**

A total of 120 children conceived by ART and 132 NC children under the age of five were included in our study. We observed that compared with NC group, ART children had lower IgA and IgG levels (*p* < 0.001), absolute lymphocytosis, high levels of active T-lymphocytes (*p* = 0.001), and pathological T-helper levels (*p* = 0.004). Therefore, the clinical presentation of respiratory diseases was lower in ART group. Children born after frozen embryo transfers showed significantly higher levels of T-cytotoxic and active T-lymphocytes compared to children born after fresh embryo transfers (*p* = 0.007 and *p* = 0.020, respectively). We utilized ordinal logistic regression to control for confounding variables such as multiple pregnancy, cesarean section, premature birth, and breastfeeding. Despite this, the significant impact of ART on immunogram parameters persisted, indicating the independent and influential nature of ART or other unaccounted factors.

## 1 Introduction

Assisted reproductive technology (ART) has evolved from an ambitious and experimental procedure into mainstream medicine and has become promising for couples suffering from infertility in the last 40 years (1). Globally, more than 12 million children are born (2), and more than 35,000 are born in Kazakhstan after infertility treatment (3). While birth outcomes and most health aspects are well documented, understanding the influence of ART on the immune system of children is still an area of ongoing research.

Current data indicate that ART-conceived children have a greater incidence of dermatological (4), respiratory (5, 6), gastrointestinal (7), and infectious (8) conditions. Some authors associate this technique with frozen embryo transfer (FET) (9). Children conceived after FET had an increased risk of developing alimentary allergy in early childhood, but no significant association was found with asthma, atopic dermatitis, and allergic rhinitis compared to naturally conceived (NC) children (10). Several systematic reviews have shown a slight increase in the risk of asthma among ART-conceived children compared to NC, though the findings regarding allergies remain limited and inconclusive (11, 12). The GUHS study reported no difference in asthma prevalence but noted greater lung volumes and less bronchial hyperreactivity in ART group (13). Additionally, cases of allergic rhinoconjunctivitis, alimentary allergy, and positive skin prick tests were more common in the ART group, while the rates of atopic dermatitis were similar to those of NC children (14). A cohort study by Wei et al. 2022 (15) demonstrated that the risk of hospitalization in ART-conceived children was 1.23 times greater than that in children in the non-ART group and was associated with infections and allergies compared to that in NC siblings.

Mice conceived via ART displayed less effective immune responses to vaccines and showed altered T-helper (Th) immune responses (16). A study by Xu et al. 2021 (17) showed no significant differences in total T cells and B cells, natural killer (NK), CD4 ^+^ T, CD8 ^+^ T, T-helper (Th)1 cells, Th17 cells, and regulatory T (T_reg_) cells among 69 ART-conceived children aged 3–6 years compared to NC (*p* > 0.05). A 2022 trial conducted by Mikheeva E.M. et al. (18) involved 82 ART infants who showed higher rates of illnesses during the first year. Changes in cellular immunity were established by T-lymphocytopenia in ART infants, and dysfunction of the humoral immune response manifested as dysimmunoglobulinemia (18).

Evidence shows that ART-conceived children tend to have immune disorders (11, 19–21). Understanding the effect of ART on the immune status of children will improve the timely diagnosis and treatment of immune disorders in the future. This study aimed to broaden the sample size compared to that of past studies and assess the impact of ART on the immune system with the inclusion of defined risk factors.

## 2 Methods

### 2.1 Study design and patients

This case-control study recruited women who had live births after fresh or frozen embryo transfer between January 2018 and September 2022 at three leading reproductive clinics in Almaty and who were receiving both paid and reimbursement programs. Donor oocytes/sperm or embryo recipients, intrauterine insemination, and surrogacy were excluded. The study population for controls included NC children.

### 2.2 Data collection

Anamnestic data were collected. Maternal data (obstetric, gynecological, and somatic history) were retrospectively extracted from medical records. The children's medical history, anthropometric data, and blood samples were collected for immunologic analysis at the pediatrician's appointment.

### 2.3 Laboratory methods

Fasting blood samples were collected during the morning (08:00–10:30) after an overnight fast of at least 8 h. The absolute lymphocyte counts and indicators of cellular (CD3, CD4, CD8, CD16, CD19, CD25, CD56, CD95, and CD3 HLA DR+) and humoral (IgM, IgA, and IgG) immunity were determined at the Scientific Center of Pediatrics and Pediatric Surgery. Immune status was investigated on a BD FACSLyric flow cytofluorimeter (USA) in the BD FACSuite Clinical v1.5 program using monoclonal antibodies against CD3, CD19, CD4, CD8, CD45, and CD56 presented in the BD Multitest 6-Color TBNK kit and single monoclonal antibodies (anti-HLA-DR). The samples were stained with monoclonal antibodies (mAb) using reagents from the manufacturer Becton Dickinson (BD) according to generally accepted recommendations. To do this, 5 µl of mAb was added to a tube with 50 µl of the sample under study, in our case, peripheral blood, mixed on a Vortex and incubated for 15 min at room temperature in the dark. After adding 1 ml of 1 × 10 BD FACS™ lysing reagent 10× Concentrate (1 ml of primary solution and 9 ml of distilled water), the mixture was vortexed and incubated for 10 min at room temperature in the dark. The mixture was centrifuged for 5 min at 300 g/min, after which the supernatant was removed. Then, 0.5 ml of BD CellWash solution was added.

### 2.4 Statistical analysis

The sample size calculation used the Lehr formula for average values, assuming a study power of 90% and a significance level of 0.01 (22). The minimum clinically significant difference in the lymphocyte content was determined based on a pilot study involving 20 children (5 with an 11.5 standard deviation). Upon inputting these values into the Lehr formula, we deduced the minimum required size for each of the compared groups. Consequently, a minimum of 112 patients was required for each group to meet the parameters set by the study.

All the statistical analyses were performed using SPSS statistical software (version 26; SPSS, Inc., USA). Quantitative data are presented as medians (Me) and interquartile ranges (IQRs) because of the nonnormal distribution of variables. Comparisons between groups were carried out by the Mann–Whitney *U*-test for nonnormally distributed continuous data, and Fisher's exact test and *χ*2 test for categorical data. Odds ratios (ORs) with 95% confidence intervals (CIs) were computed for all maternal somatic, obstetric, and postnatal care history variables for ART relative to spontaneous conception. If the frequency of occurrence of a trait in one of the groups was 0, the Haldane–Enscombe correction was used to calculate the odds ratio (OR). The calculation was performed using an online calculator: https://www.medcalc.org/calc/odds_ratio.php. Spearman's correlation test was applied to compare variables with non-normal distribution. The limit of statistical significance was *P* < 0.05. The ordinal logistic regression was used to assess the independent influence of ART with the elimination of confounders on meaningful indicators of the immunogram. Missing values were less than 1%.

### 2.5 Trial registration

The protocol was registered on ClinicalTrials.gov (NCT01369355) on October 17, 2023.

### 2.6 Ethical approval

This study complied with the Declaration of Helsinki and was approved by the local Ethics Committee of the “Scientific Center of Pediatrics and Pediatric Surgery” on April 13, 2022 (reference number: 2). Informed consent was obtained from all the legally authorized representatives of the research participants before enrollment in the trial.

## 3 Results

### 3.1 Cohort description

A total of 252 children aged 1 to 55 months who met the inclusion criteria were enrolled. The study group included 120 ART-conceived children, while the control group included 132 NC children. The characteristics of the study population are presented in Table 1.

**Table 1:** Characteristics of the study population conceived by assisted reproductive technology and naturally conceived.

	ART (*n* = 120)	NC (*n* = 132)	*P*-value
Age, months	14.5 [8–22.5]	23 [15–34]	<.001*
Gender, *n* (%)			
Female	52 (43.3)	57 (43.2)	0.981
Gestational age, weeks	38 [36.3–39]	39 [38–40]	<.001*
Child gestational age group, *n* (%)			0.002*
> 42 weeks (postterm)	89 (74.2)	116 (87.9)	p_1−3_<.001*
38–42 weeks (full-term)	1 (0.8)	7 (5.3)	p_2−3_<.001*
34–36 weeks (late preterm)	26 (21.7)	3 (2.3)	
32–34 weeks (moderately preterm)	0 (0)	5 (3.8)	
28–32 weeks (very preterm)	0 (0)	1 (0.8)	
<28 weeks (extremely preterm)	4 (3.3)	0 (0)	
Birthweight, gram	3,160 [2,695–3,600]	3,453 [3,140–3,690]	<.001*
Actual weight status, gram	10,600 [8,500–12,500]	11,450 [10,000–14,000]	<.001*
Birth height, cm	51 [46.5–54]	53 [51–54]	0.002*
Actual height status, cm	79 [73–87]	87 [78–95]	<.001*
The occurrences of respiratory infections per year	2 [1–4]	4 [2–6]	<.001*
Maternal age, years	34 [30–38]	28 [25–32]	<.001*
Paternal age, years	36 [32–41]	30 [28–35]	<.001*
Maternal BMI, kg/m^2^	22.6 [20.7–25.7]	20.8 [19.6–23.4]	<.001*
Smoking, *n* (%)	5 (4.2)	14 (10.6)	0.059
Alcohol, *n* (%)	0 (0)	8(6.1)	0.055

BMI, body mass index; ART, assisted reproductive technology; NC, natural conception.

Data are expressed as the median (interquartile range). *P*-values were determined by using the Mann–Whitney *U*-test for nonnormally distributed continuous data and the Chi-square test or Fisher's exact test for categorical data.

* Indicates statistical significance at *p* < 0.05.

Late premature infants significantly prevailed in ART group (*p* < 0.001). Based on the medical history data of the mothers (Table 2), the odds of multiple pregnancy in ART group were 9.65 times greater than in NC group (95% CI: 2.81–33.18).

**Table 2:** Comparison of somatic, obstetric, and postnatal medical histories between women who underwent assisted reproductive technology and those who conceived naturally.

	ART (*n* = 120)	NC (*n* = 132)	*P*-value	OR; 95% CI
Glomerulonephritis	0 (0)	3 (2.3)	0.217	0.15; 0.01–3.0
Systemic disease	0 (0)	5 (3.8)	0.114	0.1; 0.01–1.76
Allergy status	14 (11.7)	5 (3.8)	0.029*	3.36; 1.17–9.62
Bronchial asthma	1 (0.8)	2 (1.5)	1.000	0.55; 0.05–6.1
Arthritis	1 (0.8)	4 (3)	0.373	0.27; 0.03–2.44
Rheumatic heart disease	0 (0)	1 (0.8)	0.537	0.36; 0.01–9.02
Anemia of pregnancy	39 (32.5)	71 (53.8)	0.001*	0.41; 0.25–0.69
Gestational toxicosis	5 (4.2)	20 (15.2)	0.004*	0.24; 0.09–0.67
Eclampsia	0 (0)	1 (0.8)	0.537	0.36; 0.01–9.02
Preeclampsia	10 (8.3)	4 (3)	0.097	2.91; 0.89–9.54
GDM	3 (2.5)	2 (1.5)	0.671	1.67; 0.27–10.15
Multiple births	22 (18.3)	3 (2.3)	<.001*	9.65; 2.81–33.18
C-section	84 (70)	43 (32.6)	<.001*	4.83; 2.83–8.24
Breastfeeding till 6 months	69 (57.5)	104 (78.8)	<.001*	0.36; 0.21–0.63
Breastfeeding till 12 months	52 (47.3)	86 (65.2)	0.005*	0.48; 0.29–0.81

Data are expressed as *n* (%). *P*-values were determined by using the Chi-square test or Fisher's exact test for categorical data.

OR, odds ratio; CI, confidence interval; ART, assisted reproductive technology; NC, natural conception; GDM, gestational diabetes mellitus; C-section, s cesarean section.

* Indicates statistical significance at *p* < 0.05.

The parents of the NC group were younger than the parents of the ART group (*p* < 0.001). Additionally, the weight and BMI of the mothers were lower in the NC group (*p* = 0.002 and *p* < 0.001, respectively). For extragenital diseases in women in ART group, the odds of having an allergic disease were 3.36 times greater in ART group than in NC group (95% CI: 1.17–9.62). The obstetric history showed that ART-conceived women were 2.43 times less likely to experience anemia during pregnancy (95% CI = 0.25–0.69) and 4.17 times less likely to experience gestational toxicosis (95% CI = 0.09–0.67). For ART group, the odds of having a cesarean section (C-section) were 4.8 times greater than that for NC group (95% CI: 2.83–8.24). No significant differences were found in the odds of other maternal medical history data. The odds of breastfeeding in the first 6 months were 2.8 times lower for ART-conceived children than for NC-conceived children (95% CI: 0.21–0.63). The number of respiratory infections per year was significantly greater in NC children (*р* < 0.001).

### 3.2 Comparison of immune status between NC and ART children

Table 3 shows the results of the cellular and humoral immunity assessments in ART and NC children. The analyses were evaluated based on laboratory guidelines and categorized as falling within reference ranges, high, or low levels.

**Table 3:** Comparative assessment of immune cell profiles in children conceived by assisted reproductive technology versus those conceived naturally.

Immune indicators	*n*	Results			*P*-value	*P*-value**
		Reference values	High levels	Low levels		
Lymphocytes	ART (*n* = 117)	50 (42.7)	60 (51.3)	7 (6)	0.001*	<0.001*
	NC (*n* = 131)	73 (55.7)	39 (29.8)	19 (14.5)		
Т-lymphocytes (CD3 + CD19-)	ART (*n* = 118)	99 (83.9)	1 (0.8)	18 (15.3)	0.568	0.748
	NC (*n* = 132)	104 (78.8)	2 (1.5)	26 (19.7)		
В-lymphocytes (CD3-CD19+)	ART (*n* = 118)	77 (65.3)	38 (32.2)	3 (2.5)	0.241	0.074
	NC (*n* = 132)	93 (70.5)	32 (24.2)	7 (5.3)		
Th (CD4 + CD8-)	ART (*n* = 118)	95 (80.5)	14 (11.9)	9 (7.6)	0.004*	<0.001*
	NC (*n* = 132)	100 (75.8)	6 (4.5)	26 (19.7)		
T-cytotoxic (CD4-CD8+)	ART (*n* = 118)	50 (42.4)	46 (39)	22 (18.6)	0.260	0.048*
	NC (*n* = 132)	54 (40.9)	62 (47)	16 (12.1)		
Immunoregulatory index	ART (*n* = 118)	66 (55.9)	30 (25.4)	22 (18.6)	0.051	0.009*
	NC (*n* = 132)	74 (56.1)	20 (15.2)	38 (28.8)		
Active T-lymphocytes (CD3 + HLA-DR+)	ART (*n* = 118)	41 (34.7)	49 (41.5)	28 (23.7)	0.001*	0.001*
	NC (*n* = 132)	37 (28)	82 (62.1)	13 (9.8)		
CD3-HLA-DR+	ART (*n* = 118)	59 (50)	56 (47.5)	3 (2.5)	0.697	0.37
	NC (*n* = 132)	65 (49.2)	61 (46.2)	6 (4.5)		
CD16- CD56 + natural killer cells	ART (*n* = 118)	58 (49.2)	10 (8.5)	50 (42.4)	0.059	0.040*
	NC (*n* = 132)	64 (48.5)	24 (18.2)	44 (33.3)		
Т-NK (CD3+/CD16 + 56+)	ART (*n* = 118)	118 (100)	0 (0)	0 (0)	–	–
	NC (*n* = 132)	132 (100)	0 (0)	0 (0)		
Activation markers of lymphocytes CD95+	ART (*n* = 118)	111 (94.1)	7 (5.9)	0 (0)	0.802	0.896
	NC (*n* = 132)	123 (93.2)	9 (6.8)	0 (0)		
Activated T cells expressing IL-2 receptor alpha-chains (CD3 + CD25+)	ART (*n* = 118)	105 (89)	13 (11)	0 (0)	0.030*	0.297
	NC (*n* = 132)	104 (78.8)	28 (21.2)	0 (0)		
IgM	ART (*n* = 120)	120 (100)	0 (0)	0 (0)	0.499	0.998
	NC (*n* = 132)	130 (98.5)	2 (1.5)	0 (0)		
IgA	ART (*n* = 120)	29 (24.2)	0 (0)	91 (75.8)	<.001*	0.003*
	NC (*n* = 132)	61 (46.2)	0 (0)	71 (53.8)		
IgG	ART (*n* = 120)	73(60.8)	0(0)	47(39.2)	<.001*	0.003*
	NC (*n* = 132)	110(83.3)	0(0)	22(16.7)		

ART, assisted reproductive technology; NC, natural conception; Th, T helpers; NK, natural killer; IL, interleukin.

Data are expressed as *n* (%). *P*-values were determined by using the Chi-square test or Fisher's exact test for categorical data.

* Indicates statistical significance at *p* < 0.05.

** Adjusted for the following confounders: multiple pregnancy, childbirth (natural or cesarean section), premature birth, and breastfeeding till the age of 6 months from ordinal regression.

A comparison of the blood absolute lymphocyte count, Th (CD4 + CD8-), active T-lymphocyte (CD3 + HLA-DR+), activated T cell expressing IL-2 receptor alpha-chain (CD3 + CD25+), IgA, and IgG in the blood between the study groups revealed significant differences (*p* = 0.001, *p* = 0.004, *p* = 0.001, *p* = 0.030, *p* < 0.001, *p* < 0.001, respectively). The differences were explained by absolute lymphocytosis, increased levels of Th (CD4 + CD8-), and pathological levels of active T lymphocytes (CD3 + HLA-DR+) in ART children, a greater prevalence of absolute lymphocytopenia, increased levels of active T-lymphocytes (CD3 + HLA-DR+) and activated T cells expressing IL-2 receptor alpha chains (CD3 + CD25+) in NC children. Both groups exhibited low IgA and IgG levels; however, ART group displayed lower levels more frequently.

ART group exhibited a higher prevalence of multiple pregnancies, premature births via cesarean section, and a lack of breastfeeding—factors known to influence the development of children's immunity significantly (23–29). In our analysis, we employed ordinal logistic regression to control these confounding variables. The results indicated that the statistically significant effects of ART on the levels of lymphocytes, T-helper cells (CD4 + CD8-), active T-lymphocytes (CD3 + HLA-DR+), IgA, and IgG remained prominent (*p* < 0.001, *p* < 0.001, *p* = 0.001, *p* = 0.003, and *p* = 0.003, respectively). However, the statistically significant impact of ART on the levels of activated T-lymphocytes expressing the alpha chain of the IL-2 receptor, CD3 + CD25+, became non-significant (*p* = 0.299). Additionally, we observed that the statistically significant impact of ART on the levels of T-cytotoxic cells (CD4-CD8+), immunoregulatory index, and CD16- CD56 + natural killer cells has emerged (*p* = 0.048, *p* = 0.009, and *p* = 0.040, respectively).

**Figure 1:**
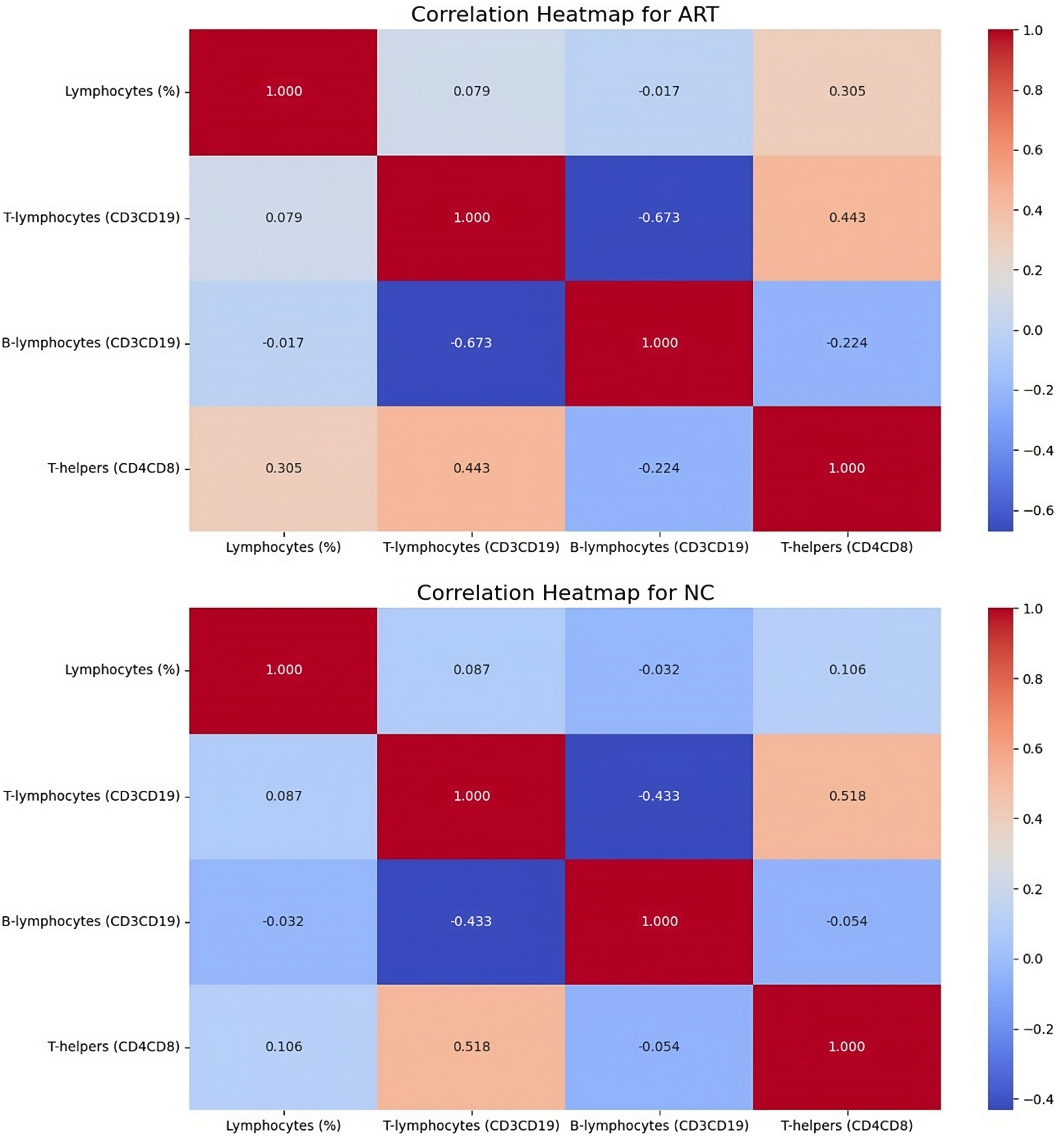
Correlation analysis of immune markers in children conceived by assisted reproductive technology and naturally conceived. **(A)** Correlation heatmap for ART-conceived children. **(B)** Correlation heatmap for NC children.

Figure 1 describes the correlation between immune markers such as lymphocytes (%), T-lymphocytes (CD3 + CD19-) (%), B-lymphocytes (CD3-CD19+) (%), and Th (CD4 + CD8-) (%) in ART and NC groups.

Both in ART and NC groups, T-lymphocytes (CD3 + CD19-) showed a significant moderate positive correlation with Th (*p* < 0.001) and a significant moderate negative correlation with B lymphocytes (*p* < 0.001). In ART group Th showed a significant weak positive correlation with lymphocytes (*p* = 0.001) and a significant weak negative correlation with B lymphocytes (*p* = 0.015).

### 3.3 Comparison of immune status between FET group and fresh-ET group

We assessed the immune status of children born after FET or fresh embryo transfer (Fresh-ET) (Table 4). Overall, 24.2% (*n* = 29) of the children conceived using Fresh-ET, and 75.8% conceived using FET (*n* = 91).

**Table 4:** Comparative assessment of immune cell profiles in children conceived by frozen vs. fresh embryo transfer.

Immune indicators	*n*	Results of assessment of immune cell count			*P*-value	*P*-value**
		Reference values	High levels	Low levels		
Lymphocytes	Fresh-ET (*n* = 29)	13 (44.8)	12 (41.4)	4 (13.8)	0.095	0.089
	FET (*n* = 88)	37 (42)	48 (54.5)	3 (3.4)		
Т-lymphocytes (CD3 + CD19-)	Fresh-ET (*n* = 29)	26 (89.7)	0 (0)	3 (10.3)	0.581	0.516
	FET (*n* = 89)	73 (82)	1 (1.1)	15 (16.9)		
В-lymphocytes (CD3-CD19+)	Fresh-ET (*n* = 29)	20 (69)	8 (27.6)	1 (3.4)	0.795	0.399
	FET (*n* = 89)	57 (64)	30 (33.7)	2 (2.2)		
Th (CD4 + CD8-)	Fresh-ET (*n* = 29)	20 (69)	8 (27.6)	1 (3.4)	0.009*	0.009*
	FET (*n* = 89)	75 (84.3)	6 (6.7)	8 (9)		
T-cytotoxic (CD4-CD8+)	Fresh-ET (*n* = 29)	11 (37.9)	7 (24.1)	11 (37.9)	0.007*	0.009*
	FET (*n* = 89)	39 (43.8)	39 (43.8)	11 (12.4)		
Immunoregulatory index	Fresh-ET (*n* = 29)	19 (65.5)	9 (31)	1 (3.4)	0.053	0.189
	FET (*n* = 89)	47 (52.8)	21 (23.6)	21 (23.6)		
Active T-lymphocytes (CD3 + HLA-DR+)	Fresh-ET (*n* = 29)	12 (41.4)	6 (20.7)	11 (37.9)	0.020*	0.007*
	FET (*n* = 89)	29 (32.6)	43 (48.3)	17 (19.1)		
CD3-HLA-DR+	Fresh-ET (*n* = 29)	19 (65.5)	9 (31)	1 (3.4)	0.125	0.048*
	FET (*n* = 89)	40 (44.9)	47 (52.8)	2 (2.2)		
CD16- CD56 + natural killer cells	Fresh-ET (*n* = 29)	10 (34.5)	3 (10.3)	16 (55.2)	0.189	0.184
	FET (*n* = 89)	48 (53.9)	7 (7.9)	34 (38.2)		
Т-NK (CD3+/CD16 + 56+)	Fresh-ET (*n* = 29)	29 (100)	0 (0)	0 (0)	–	–
	FET (*n* = 89)	89 (100)	0 (0)	0 (0)		
Activation markers of lymphocytes CD95+	Fresh-ET (*n* = 29)	29 (100)	0 (0)	0 (0)	0.192	–
	FET (*n* = 89)	82 (92.1)	7 (7.9)	0 (0)		
Activated T cells expressing IL-2 receptor alpha-chains (CD3 + CD25+)	Fresh-ET (*n* = 29)	26 (89.7)	3 (10.3)	0 (0)	1.000	0.773
	FET (*n* = 89)	79 (88.8)	10 (11.2)	0 (0)		
IgM	Fresh-ET (*n* = 29)	29 (100)	0 (0)	0 (0)	–	–
	FET (*n* = 91)	91 (100)	0 (0)	0 (0)		
IgA	Fresh-ET (*n* = 29)	3 (10.3)	0 (0)	26 (89.7)	0.046*	0.022*
	FET (*n* = 91)	26 (28.6)	0 (0)	65 (71.4)		
IgG	Fresh-ET (*n* = 29)	14 (48.3)	0(0)	15 (51.7)	0.112	0.118
	FET (*n* = 91)	59 (64.8)	0(0)	32 (35.2)		

FET, frozen embryo transfer; Fresh-ET, fresh embryo transfer; NK, natural killer; Th, T helper; IL, interleukin.

Data are expressed as *n* (%). *P* values were determined by using the Chi-square test or Fisher's exact test for categorical data.

* Indicates statistical significance at *p* < 0.05.

** Adjusted for the following confounders: multiple pregnancy, childbirth (natural or cesarean section), premature birth, and breastfeeding till the age of 6 months from ordinal regression.

The levels of T-cytotoxic (CD4-CD8+) and active T-lymphocytes (CD3 + HLA-DR+) were significantly greater in FET group than in the Fresh-ET group (*p* = 0.007 and *p* = 0.020, respectively). The level of Th (CD4 + CD8-) was higher in Fresh-ET group (*p* = 0.009), while the level of IgA was significantly lower in both groups. In our analysis using ordinal logistic regression to mitigate confounding factors such as multiple pregnancy, cesarean section, premature birth, and breastfeeding, observed a statistically significant influence of Fresh-ET on the levels of T-helper cells (CD4 + CD8-), T-cytotoxic (CD4-CD8+), active T-lymphocytes (CD3 + HLA-DR+), CD3-HLA-DR+, and IgA (*p* = 0.009, *p* = 0.009, *p* = 0.007, *p* = 0.048, and *p* = 0.022, respectively).

## 4 Discussion

The key findings of this case-control study are as follows: (i) a greater incidence of allergic diseases was observed in ART mothers; (ii) a statistically significant difference in immune parameters was found in ART children; and (iii) a statistically significant difference in immune parameters was found in FET group.

The data consistently showed that women who underwent ART were diagnosed more often with allergic diseases (95% CI: 1.17–9.62), confirming the results of several studies on the relationship between allergic diseases in women and subfertility, thereby increasing the risk of immune pathologies in offspring (21, 30, 31). Maternal allergies can trigger the development of allergies in offspring, either through inherited allergic predispositions or by creating an environment that promotes allergic disorders (32). Having a parental history of allergic disease increases a child's risk of developing allergies (33), with the combined influence of both parents posing a greater risk than either parent alone (34). Certain genes that regulate the T-cell response have been linked to the predisposition for developing allergic disorders (35). Furthermore, it has been found that maternal drug allergies are associated with a higher risk of long-term infectious hospitalizations in offspring, suggesting a potential inheritance of immune dysfunctions from mother to child (36).

Low levels of IgA and IgG were observed in both groups (*p* < 0.001 and *p* < 0.001, respectively). However, lower IgA and IgG levels were more common in ART group which confirmed the data of Mikheeva E.M. et al. 2022 (18). Notably, IgG deficiency, which is involved in the secondary immune response, reduces the effectiveness of vaccination, which confirms the results of a study of mice conceived using ART (16). Absolute lymphocytosis and increased Th levels were significantly more common in ART group (*p* = 0.001 and *p* = 0.004, respectively). Furthermore, a statistically significant correlation between these variables was established (*p* = 0.001). Additionally, a notable negative correlation between B-lymphocytes and Th levels was identified (*p* < 0.001), indicating that B cell dysfunction contributed to a diminished immunoglobulin response. Th cells are a diverse group of cells essential for adaptive immunity (37). They have a direct and indirect impact on nearly every aspect of the immune response. This population includes effector cells, which are designed to defend against infections, and Tregs, which help prevent excessive reactions to both self-antigens and harmful external antigens. Th cells play a crucial role in assisting B cells in generating antibodies during the initial immune response (humoral immunity). Changes in Th status can significantly contribute to the development of autoimmune, inflammatory, and allergic disorders in ART offspring in the future (37). Reduced levels of IgG and Th may significantly contribute to the diminished efficacy of vaccination in ART-conceived children, which warrants further investigation to elucidate the underlying mechanisms involved. Both groups showed high levels of active T-lymphocytes and activated T cells expressing IL-2 receptor alpha chains (CD3 + CD25+), but 23.7% of children in ART group had decreased levels of active T-lymphocytes (CD3 + HLA-DR+). When comparing the immune status of a group of children conceived using FET and Fresh-ET, the levels of T-cytotoxic (CD4-CD8+) and active T-lymphocytes (CD3 + HLA-DR+) were significantly greater in FET group and lower in Fresh-ET group (*p* = 0.007 and *p* = 0.020, respectively). The Th level was greater in Fresh-ET group (*p* = 0.009), while the IgA level was significantly lower in both groups (*p* = 0.046). The activation of cellular immunity was revealed, which indicated more frequent infectious diseases and respiratory diseases in FET group than in Fresh-ET group, confirming the findings of previous studies (9). Our data on the occurrence of respiratory infections per year in ART-conceived children differ from those in the study by Mikheeva E.M. et al. (18), where ART-conceived children were classified as frequently ill. According to our study, the occurrence of respiratory infections per year was lower in ART group than in the control group, which indicates a reduced immune response in this cohort.

Previous studies on the immune status of ART-conceived children failed to consider risk factors other than ART use in their analysis. This study factored in medical history data and revealed that ART-conceived children were mostly born by C-section (95% CI: 2.83–8.24) and were significantly less likely to be breastfed for at least the first 6 months (95% CI: 0.21–0.63). In addition, ART-conceived children were more likely to be born prematurely compared to NC group (*p* < 0.001).

The development of the gut microbiota at an early age is crucial for the balanced preparation of the immune system, which occurs in the early stages of life in the so-called “window of opportunity” (23). C-section birth is associated with adverse effects on immune development (24), particularly in the inflammatory response to infection and in cells involved in the allergic response, during infancy and early childhood (25, 26). Prematurity is associated with a high risk of dysbiosis and delayed colonization with decreased diversity (27). Furthermore, a decrease in breastfeeding success exacerbates changes in the early development of the gut microbiota (28, 29).

In our analysis, we utilized ordinal logistic regression to eliminate confounding variables and accurately assess the impact of multiple pregnancy, cesarean section, premature birth, and breastfeeding. Despite our rigorous approach, the statistically significant impact of ART on the immunogram parameters remained, underscoring the independent and influential nature of ART or other unaccounted factors.

It is biologically conceivable that ART could contribute to health issues in children. One theory posits that the mechanical and hormonal intervention of gametes and embryos might trigger epigenetic modifications, which could impact the immune system and lower disease resistance. ART procedures take place during a critical stage of development that is vital for epigenetic reprogramming. As a result, technical interventions during this delicate period may lead to a modified epigenetic profile of the conceived child (38). Several studies have observed altered epigenetic characteristics in gametes from infertile couples, raising concerns about a higher risk of imprinting disorders and somatic epigenetic alterations in children conceived via ART (39–41). Changes in gene expression related to immune response have also been observed in the placentas of individuals undergoing IVF, suggesting a possible effect on the immune response of their offspring (42). Additionally, pregnancies conceived using ART have been associated with a greater risk of tumor development, which may indicate an increased immune tolerance to tumor antigens (43). The application of ART involves administering sex hormones. Even normal hormonal changes during pregnancy can trigger an inflammatory response, along with metabolic and endocrine alterations (44). Current evidence suggests that women who conceive through IVF are more likely to develop gestational diabetes mellitus compared to those who conceive spontaneously. This correlation remains significant even after accounting for factors such as maternal age, gestational age, and parity (45, 46). Moreover, gestational diabetes mellitus can lead to immune dysregulation, inflammation, changes in the microbiota, and allergic diseases in the offspring due to epigenetic changes brought about by a hyperglycemic environment in the uterus (47, 48). These research findings and clinical observations suggest that ART could affect the immune profile of the offspring.

To enhance the immune system health of ART-conceived children and improve the efficacy of vaccinations, medical leaders in Kazakhstan are advised to incorporate consultations with immunologists to assess the immune status within the first year of life. Continuous monitoring of the immune system of ART children is also recommended to promptly address any changes that may occur.

### 4.1 Limitations

First, the size of the study group does not reflect the wider population. Lehr's formula proposed a minimum number of patients sufficient to compare immune parameters between ART-conceived and NC children. Second, our study is limited by a short follow-up period due to the complexity and cost of longitudinal studies, which limits the ability to assess the long-term effects of ART procedures on the immune system of offspring. To enhance this understanding, future research must prioritize this consideration. Third, we assessed a few risk factors, excluding others, such as infertility itself, drugs used for ovulation induction, placental pathology, differences in lifestyle choices, other diseases, and high levels of anxiety in couples. Fourth, in addition to considered confounders (multiple pregnancy, cesarean section, premature birth, and breastfeeding), there are significant differences in other general characteristics (e.g., gestational age, birth weight, and others), that could affect the reliability of the study's findings, which would be good to take into account in future research. Fifth, significant confounding factors, including the type of ART procedures, as well as the protocols for ovarian stimulation, were not sufficiently controlled in the current study. These factors may considerably influence the immune outcomes of the offspring and should be addressed in future research to enhance the accuracy and reliability of the findings. Sixth, this study included only a sample from Kazakhstan. It is necessary to expand the geography of studies to increase the sample size and increase the applicability of findings to other ethnic groups. Thus, longitudinal studies should be conducted considering various risk factors for immune diseases to obtain a clear overview of the long-term effects of ART on the immune status of offspring.

## 5 Conclusion

Despite the limited number of studies on the immune system of ART-conceived offspring, our study confirmed the altered immune profiles in ART-conceived children. Specifically, they tend to have lower levels of IgA and IgG, absolute lymphocytosis, and increased Th cell counts which can result in a poor immune response and vaccine effectiveness. However, considering the analysis of risk factors, further research is needed to comprehensively distinguish between the role of ART treatment and parental factors in determining the immune outcomes of offspring.

## Data Availability

The raw data supporting the conclusions of this article will be made available by the authors, without undue reservation.
