# Supplementary material for: Immune profiling of ART-conceived children in Kazakhstan: a case-control study
Source: Front Pediatr. 2024 Nov 22;12:1447956. doi: 10.3389/fped.2024.1447956 (PMC11620871; doi:10.3389/fped.2024.1447956)
Supplement: Supplementary file 1 [file Table1.docx]

Supplementary Material

# Supplementary Data

An additional table 1 shows the median levels of immune indicators in the peripheral blood in ART and NC children.

Additional file 1: Table S1. Immunological status in ART and NC children.

| The levels of immune indicators in peripheral blood | Groups | | | | P value |
| --- | --- | --- | --- | --- | --- |
|  | ART (n=120) | | NC (n=132) | |  |
|  | Me [IQR] | min-max | Me [IQR] | min-max |  |
| Lymphocytes (%) | 62.4 [54.2-70.0] | 2.1-84.3 | 53.7 [41.9-60.25] | 3.9-77.7 | <0.001* |
| T-lymphocytes (CD3+CD19-) (%) | 67.3 [63.3-71.0] | 45.9-81.2 | 66.8 [62.4-72.2] | 25.9-82.8 | 0.823 |
| B-lymphocytes (CD3-CD19+) (%) | 20.1 [17.4-25.8] | 4.5-43.8 | 18.9 [15.1-22.8] | 2.7-33.7 | 0.010* |
| Th (CD4+CD8-) (%) | 38.4 [34.6-44.3] | 19.1-61.7 | 36.9 [31.4-42.1] | 20.4-57.8 | 0.008* |
| T-cytotoxic (CD4-CD8+) (%) | 23.1 [19.0-28.0] | 11.4-44.3 | 24.4 [20.6-29.7] | 11.8-44.3 | 0.052 |
| Immunoregulatory index | 1.7 [1.3-2.3] | 0.6-4.5 | 1.5 [1.1-1.9] | 0.5-4.1 | 0.008* |
| Active T-lymphocytes (CD3+HLA-DR+) (%) | 8.6 [5.1-14.0] | 1.6-31.9 | 11.5 [7.7-14.6] | 2.8-40.0 | 0.002* |
| CD3-HLA-DR+ (%) | 22.65 [19.50-27.80] | 4.5-45.2 | 22.55 [18.20-26.15] | 2.9-35.2 | 0.147 |
| CD16- CD56+ natural killer cells (%) | 8.3 [5.8-12.1] | 1.4-23.2 | 9.9 [6.7-15.7] | 1.1-37.5 | 0.007* |
| Т-NK (CD3+/CD16+56+) (%) | 1.3 [0.8-1.7] | 0.2-9.3 | 1.4 [1.0-2.0] | 0.4-6.5 | 0.089 |
| Activated T cells expressing IL-2 receptor alpha-chains. CD3+CD25+ (%) | 6.8 [4.0-8.1] | 0.2-12.2 | 7.9 [6.8-9.6] | 0.4-13.1 | <0.001* |
| Activation markers of lymphocytes. CD95+ (%) | 3.2 [1.9-5.1] | 0.1-74 | 5.5 [3.9-6.8] | 0.6-14.8 | <0.001* |
| IgM level (%) | 0.69 [0.47-0.98] | 0.25-2.53 | 0.92 [0.62-1.20] | 0.25-3.28 | <0.001* |
| IgA level (%) | 0.38 [0.25-0.60] | 0.25-1.18 | 0.60 [0.38-0.82] | 0.25-1.52 | <0.001* |
| IgG level (%) | 6.42 [4.21-8.1] | 3.20-17.2 | 7.77 [6.04-9.55] | 3.20-16.0 | <0.001* |

*Note:*

*Abbreviations: NK - natural killer; IL – interleukin; Th – T helpers; ART - assisted reproductive technologies; NC - natural conception.*

*Data are expressed as the median (interquartile range). P values were determined by using the Mann–Whitney U test for non-normally distributed continuous data.*

** indicates statistical significance at p < 0.05.*

We used the standards applied in our laboratory to assess the results of the laboratory analysis [see Additional file 2].

Additional file 2: Table S2. Laboratory test standards used in the study

| **Indicators** | **Reference range** |
| --- | --- |
| Т-lymphocytes (CD3+CD19-) | 60-80%(0.800-2.200) |
| В-lymphocytes (CD3-CD19+) | 10-23%(0.100-0.600) |
| Th (CD4+CD8-) | 30-50%(0.400-1.100) |
| T-cytotoxic (CD4-CD8+) | 18-25%(0.300-0.700) |
| Immunoregulatory index | 1.2-2.2 |
| Active T-lymphocytes (CD3+HLA-DR+) | 5.0-10.0%(0.04-0.3) |
| CD3-HLA-DR+ | 10-23%(0.11-0.60) |
| CD16- CD56+ natural killer cells | 8-17%(0.10-0.60) |
| Т-NK (CD3+/CD16+56+) | <10% |
| Activation markers of lymphocytes CD95+ | <10% |
| Activated T cells expressing IL-2 receptor alpha-chains CD3+CD25+ | <10% |
| IgM | 0,22-2,93 g/l |
| IgA | 0,63-4,84 g/l |
| IgG | 5,40-18,22 g/l |

*Note:*

*Abbreviations: Th – T helpers; NK - natural killer; IL – interleukin.*

An additional table 3 shows the median of the levels of immune indicators in peripheral blood in FET and Fresh-ET groups.

Additional file 3: Table S3. Immunological status in FET and Fresh-ET groups.

| The levels of immune indicators in peripheral blood | Groups | | | | P value |
| --- | --- | --- | --- | --- | --- |
|  | Fresh-ET | | FET | |  |
|  | Me [IQR] | min-max | Me [IQR] | min-max |  |
| Lymphocytes (%) | 65.0 [62.4-72.0] | 4.0-82.5 | 64.0 [53.5-69.1] | 2.1-84.3 | 0.058 |
| T-lymphocytes (CD3+CD19-) (%) | 69.3 [64.7-73.9] | 47.6-79.3 | 67.0 [61.7-70.1] | 45.9-81.2 | 0.077 |
| B-lymphocytes (CD3-CD19+) (%) | 19.7 [18.1-23.1] | 4.5-36.1 | 20.5 [17.4-26.0] | 5.8-43.8 | 0.719 |
| Th (CD4+CD8-) (%) | 43.8 [38.0-50.3] | 28.8-61.7 | 37.9 [33.8-42.4] | 19.1-58.0 | 0.001* |
| T-cytotoxic (CD4-CD8+) (%) | 21.0 [16.3-24.4] | 12.4-34.5 | 24.2 [19.5-28.5] | 11.4-44.3 | 0.018* |
| Immunoregulatory index | 2.1 [1.6-3.2] | 0.9-4.5 | 1.7 [1.2-2.2] | 0.6-4.0 | 0.006* |
| Active T-lymphocytes (CD3+HLA-DR+) (%) | 6.9 [3.7-8.7] | 1.6-21.9 | 9.8 [5.8-15.0] | 1.9-31.9 | 0.003* |
| CD3-HLA-DR+ (%) | 21.6 [18.9-26.6] | 7.3-37.5 | 23.9 [19.7-27.8] | 4.5-45.2 | 0.237 |
| CD16- CD56+ natural killer cells (%) | 6.7 [4.6-12.9] | 1.4-23.2 | 8.3 [6.2-12.1] | 2.7-22.4 | 0.152 |
| Т-NK (CD3+/CD16+56+) (%) | 1.4 [1.0-1.6] | 0.2-4.9 | 1.2 [0.8-1.8] | 0.2-9.3 | 0.752 |
| Activated T cells expressing IL-2 receptor alpha-chains. CD3+CD25+ (%) | 7.0 [4.7-7.8] | 1.7-11.8 | 6.8 [3.1-8.2] | 0.2-12.2 | 0.418 |
| Activation markers of lymphocytes. CD95+ (%) | 2.5 [1.9-3.7] | 0.1-8.9 | 3.5 [2.2-5.5] | 0.1-74 | 0.086 |
| IgM level (%) | 0.6 [0.4-0.8] | 0.3-1.7 | 0.7 [0.5-1.0] | 0.3-2.5 | 0.205 |
| IgA level (%) | 0.4 [0.3-0.4] | 0.3-1.1 | 0.4 [0.3-0.7] | 0.3-1.2 | 0.074 |
| IgG level (%) | 5.2 [3.3-7.6] | 3.2-11.0 | 6.5 [4.5-8.2] | 3.2-17.2 | 0.073 |

*Note:*

*Abbreviations: FET - frozen embryo transfer; Fresh-ET fresh – fresh embryo transfer; Th – T helpers; NK - natural killer; IL – interleukin.*

*Data are expressed as the median (interquartile range). P values were determined by using the Mann–Whitney U test for non-normally distributed continuous data.*

** indicates statistical significance at p < 0.05.*
